# Effect of Insulin on ACE2 Activity and Kidney Function in the Non-Obese Diabetic Mouse

**DOI:** 10.1371/journal.pone.0084683

**Published:** 2014-01-06

**Authors:** Marta Riera, Eva Márquez, Sergi Clotet, Javier Gimeno, Heleia Roca-Ho, Josep Lloreta, Nuria Juanpere, Daniel Batlle, Julio Pascual, María José Soler

**Affiliations:** 1 Department of Nephrology, Hospital del Mar-IMIM, Barcelona, Spain; 2 Department of Pathology, Hospital del Mar-IMIM, Barcelona, Spain; 3 Division of Nephrology and Hypertension, Department of Medicine, The Feinberg School of Medicine, Northwestern University, Chicago, Illinois, United States of America; University of Michigan Medical School, United States of America

## Abstract

We studied the non-obese diabetic (NOD) mice model because it develops autoimmune diabetes that resembles human type 1 diabetes. In diabetic mice, urinary albumin excretion (UAE) was ten-fold increased at an “early stage” of diabetes, and twenty-fold increased at a “later stage” (21 and 40 days, respectively after diabetes diagnosis) as compared to non-obese resistant controls. In NOD Diabetic mice, glomerular enlargement, increased glomerular filtration rate (GFR) and increased blood pressure were observed in the early stage. In the late stage, NOD Diabetic mice developed mesangial expansion and reduced podocyte number. Circulating and urine ACE2 activity were markedly increased both, early and late in Diabetic mice. Insulin administration prevented albuminuria, markedly reduced GFR, blood pressure, and glomerular enlargement in the early stage; and prevented mesangial expansion and the reduced podocyte number in the late stage of diabetes. The increase in serum and urine ACE2 activity was normalized by insulin administration at the early and late stages of diabetes in Diabetic mice. We conclude that the Diabetic mice develops features of early kidney disease, including albuminuria and a marked increase in GFR. ACE2 activity is increased starting at an early stage in both serum and urine. Moreover, these alterations can be completely prevented by the chronic administration of insulin.

## Introduction

Early diabetic nephropathy both in humans and rodent models is characterized by an increased glomerular filtration rate (GFR), albuminuria and renal enlargement [1], [2], [3], [4]. In type 1 diabetic patients, strict glycemic control has been shown to improve both abnormal renal function and reduce kidney volume, demonstrating that in human renal enlargement can revert to normal despite established diabetes and early evidence of nephropathy [5]. The study of kidney involvement in rodent models of diabetes has relied heavily on inducing diabetes using streptozotocin (STZ), a drug that is also nephrotoxic [6]. While this approach provides valuable information to examine the effects of hyperglycemia on the kidney, it is limited by variable responses attributable, in part, to the doses used and the duration of exposure [6]. Accordingly, there is a need for rodent models that better recapitulate the development and phenotypic features of diabetic nephropathy in type 1 and type 2 diabetes [7], [8]. Non-obese diabetic (NOD) mice is a model that develops spontaneous autoimmune diabetes, which shares many similarities to autoimmune or type 1 diabetes in human subjects, including the presence of pancreatic specific autoantibodies, autoreactive CD4+ and CD8+ T cells, and genetic linkage to disease similar to that found in humans [9]. Although previous studies have shown the development of albuminuria in the NOD mice [10], [11], there is a paucity of information on GFR, blood pressure and detailed kidney pathology findings in this model that so closely resembles human type 1 diabetes. Moreover, the effect of chronic insulin administration on the evolution of kidney disease development in NOD diabetic mice has not been well characterized.

The renin-angiotensin system (RAS) has been implicated in the pathogenesis of diabetic kidney disease [12]. In the last decade, an angiotensin converting enzyme related carboxypeptidase, angiotensin converting enzyme (ACE)2 has been identified in human and differs from ACE in that it preferentially removes carboxy-terminal hydrophobic or basic amino acids [13], [14]. ACE2 activity may counterbalance the angiotensin (Ang) II promoting effects of ACE by preventing Ang II accumulation in tissues where ACE2 and ACE are both expressed [15], [16]. Serum ACE2 activity was recently reported to be increased in male and female patients at late stages of type 1 diabetes with altered kidney function or other vascular complications such as cardiovascular heart disease [17]. In the present study, we examined serum and urine ACE2 activity in a model of diabetic kidney disease, the NOD mouse, and hypothesized that the activity of this enzyme is increased in an early stage of the disease. In addition, we also studied the effect of insulin administration to achieve tight glycemic control on preventing diabetic renal alterations and the increase of ACE2 activity levels in NOD diabetic mice.

## Materials and Methods

### Animal Model and Experimental Groups

Female NOD/ShiLtJ and control female NOR/LtJ mice were used for the study (Jackson Laboratory, Bar Harbor ME, USA). Female mice only were used as the development of diabetes is more predictable in female than in male NOD mice [18]. The mice were housed in metabolic cages with *ad libitum* access to mice chow and water. Animals were maintained under Specific Pathogen Free conditions in ventilated microisolators. The Ethical Committee of Animal Experimentation of the Barcelona Biomedical Research Park (CEEA-PRBB) approved this study.

Fasting blood samples from saphenous vein were obtained and used for glucose level determination using the ACCU-CHEK Compact® (Roche). Mice had their blood glucose levels determined every two weeks starting at 10 weeks of age. Female NOD mice were considered diabetic when a glucose blood level higher than 250 mg/dl was first detected. NOD mice that did not develop diabetes were excluded from the study (25%). Diabetic mice were studied in two groups: “early stage” and “late stage” of diabetes. The “early stage” included 16 animals that were studied 21 days following the diabetes diagnosis and 8 animals from de non-diabetic control strain NOR. The “late stage” included 14 animals that were studied 40 days following the diabetes diagnosis and 8 control animals. NOD diabetic mice from early and late stage were randomly assigned to two groups, with or without blood glucose levels control by insulin. Insulin pellets (∼0.1 U/24 hr/pellet, LinBit, LinShin Canada Inc) were subcutaneously implanted under anesthesia with ketamine and medetomidine, for diabetes treatment. After surgery, atipamezol was injected to reverse the effects of medetomidine.

Animals were monitored for body weight and blood glucose levels weekly and then sacrificed at 21 and 40 days following diabetes diagnosis for the early and late stage group, respectively. There were no differences in age at sacrifice (Table 1). Animals were anesthetized using pentobarbital sodium injection. After obtaining blood samples by cardiac puncture, mice were perfused briefly with ice cold Phosphate Buffered Saline (PBS) by transcardiac puncture to flush out blood. Kidneys were then removed, weighted and processed for several purposes. Half of one kidney from each mouse was fixed in formalin solution 10%, neutral buffered, and processed for paraffin embedding according to standard procedures. A very small proportion of the kidney was fixed in glutaraldehyde for electronic microsopy analysis. The rest of renal tissue was snap frozen in liquid nitrogen kept at −80°C until its use.

**Table 1:** Animal characteristics for NOD diabetic mice (Diabetic), NOD diabetic mice treated with insulin (Diabetic+INS) and NOR control mice (Control).

	Early stage of diabetes			Late stage of diabetes		
	Control	Diabetic	Diabetic+INS	Control	Diabetic	Diabetic+INS
*N*	8	9	7	8	8	6
Age (weeks)	21.6±0.20	21.49±0.60	20.53±0.77	21. 7±0.16	20.8±0.97	22.43±1.03
Blood Glucose (mg/dL)	112.0±4.9	571.1±20.3*^‡^	122.7±27.0	138.8±4.82	560.6±32.9*	97.7±9.48
Body weight (g)	27.2±1.12	24.9±1.03	24.4±0.71	28.67±0.72	17.2±0.49*^‡^	24.3±1.03^†^
Right kidney weight (mg)	150.0±6.55	253.3±14.2*^‡^	215.7±11.7^†^	147.5±9.77	136.3±9.62^‡^	188.3±15.6^†^
Ratio kidney/body weight	0.010±0.0005	0.020±0.0006*^‡^	0.017±0.0006^†^	0.010±0.0006	0.015±0.009*	0.015±0.022^†^

Data are expressed as means±s.e.

p≤0.05*Diabetic vs Control.

p≤0.05^‡^Diabetic vs. Diabetic +INS.

p≤0.05^†^Control vs. Diabetic+INS.

### Blood Pressure Measurements

The tail cuff method was used to measure blood pressure (CODA, Kent Scientific Corporation). Values were obtained from conscious-trained mice on five consecutive morning sessions. Results are expressed in mmHg.

### Measurements of Urinary Albumin Excretion

Urinary excretion of albumin was determined using the albumin-to-creatinine ratio (UAE) on morning spot urine collections. Urinary albumin and creatinine levels were measured by ELISA (Albuwell M, Exocell, Philadelphia, PA) and a creatinine assay kit (Creatinine Companion, Exocell) [15].

### Measurements of Glomerular Filtration Rate in Unconscious Mice

The GFR was measured using clearance kinetics of plasma FITC-inulin after a single bolus injection [19]. Dialyzed 5% FITC-inulin was injected into the tail vein, followed by collection of 30 µl of saphenous vein blood at 3, 7, 10, 15, 35, 55, and 75 min after the injection in anesthetized mice. Plasma fluorescence was determined at each time point using a Tecan Infinite 200 reader (TECAN Instruments) at an excitation wavelength of 485 nm and an emission wavelength of 538 nm. The decay in plasma fluorescence levels was fit to a two-phase exponential decay curve using nonlinear regression (GraphPad Prism, GraphPad Software, San Diego, CA). GFR was calculated as previously described and values were expressed as microliters of FITC-inulin cleared per minute [20].

### Renal Morphology

Paraffin blocks were cut at 4 µm and desparaffinized in xylene and rehydrated through graded alcohols. Kidney slices were stained using hematoxylin–eosin and Periodic Acid Schiff stain (PAS) in order to analyze the presence of renal lesions related to diabetes, or any other morphological changes. Tissues were then examined by three blinded observers.

### Estimation of Glomerular Volume, Mesangial Index, and Podocyte Number

The glomerular volume was calculated using a computer-based image-analysis system (Image J) as previously described [15]. The detection of mesangial matrix accumulation in vivo was performed by PAS staining of paraformaldehyde-fixed kidneys. Twenty glomeruli were randomly selected from each animal, and the extent of extracellular mesangial matrix was identified by PAS-positive material in the mesangium by using a computer image analysis system and factored by the glomerular tuft area (Image J, NIH) as previously described by members of the Animal Models of Diabetic Complication Consortium. Podocytes were identified using a rabbit polyclonal antibody directed against Wilm’s Tumor 1 (WT-1, Santa Cruz Biotechnology, Inc, Santa Cruz, CA), a podocyte-specific marker. To assess podocyte number in glomerular tuft, a semiquantitative analysis of the WT-1 positive cells was performed based as previously described [21], [22]. Sections were then examined by three different masked observers, who counted the cells within 20 glomeruli. The WT-1 positive cells were counted as podocyte and the hematoxylin-counterstained nuclei were counted as glomerular cells of other types. The data were expressed as the percentage of podocytes within the total glomerular cells.

### Electron Microscopy

Kidney cortex was cut into 1-mm^3^ blocks and fixed in 2.5% glutaraldehyde in 0.1M sodium cacodylatebuffer at 4°C overnight. Tissue blocks were post-fixed with 2% osmium tetroxide in 0.1M sodium cacodylate buffer for 1 h and dehydrated in graded ethanol. Blocks were infiltrated in epon/araldite resin, transferred into BEEM capsules with resin and polymerized at 60°C for 24 h. The 90 nm sections were stained with uranyl acetate and lead citrate, and photographed using a JEOL 1220 electron microscope at low (×1950) and high (×5800) magnification [15]. Three glomeruli were studied following and adapted method from the previously reported by MacLeod et al. for mesangial volume fraction estimation [23]. Briefly, a regular grid consisting of a tessellation of 8 fine points per coarse point was placed in random fashion in the picture at low magnification using the image J program. Coarse points landing on the tuft, defined as capillaries and mesangium contained within a minimal string polygon were counted (CP). Fine points landing on mesangium, consisting of mesangial matrix, mesangial basement membrane-like material and mesangial cellular components were also counted (FP). Mesangial volume fraction was estimated as FP mesangium/CP tuft x8.

Glomerular basement membrane (GBM) width was measured by the orthogonal intercept method. Four electron micrographs that were taken of random capillaries around the periphery of three glomeruli, using the technique previously described by Jensen et al. with modifications. A grid with eighteen evenly spaced intersecting lines (nine horizontal and nine vertical) was placed over a photomicrograph by Image J software. GBM measurements were made at each point that a line on the grid intercepts an endothelial-GBM interface. All intercepts so drawn must be orthogonal to the chosen aspect of the membrane. For each animal 250–350 measurements were achieved. The arithmetic mean of these orthogonal intercept lengths, multiplied by π/4, provides an estimate of GBM [24], [25].

### Serum and Urine ACE2 Enzymatic Activity

The ACE2 fluorescent enzymatic assay protocol was performed as previously described with modifications, using a specific ACE2 quenched fluorogenic substrate (Mca-Ala-Pro-Lys(Dnp)-OH, Enzo Life Sciences) [26], [27]. Briefly, 5 µL of serum or 2 µL of diluted urine (1∶10) samples were incubated with a buffer (100 mM Tris-HCl, 600 mM NaCl, 10 µM ZnCl2, pH 7.5) in the presence of protease inhibitors, including 100 µM captopril, 5 µM amastatin, 5 µM bestatin (all from Sigma-Aldrich), and 10 µM Z-Pro-prolinal (Enzo Life Sciences). Samples were incubated with 20 µM quenched fluorogenic substrate in reaction buffer (final reaction volume 100 µL) at 37°C. Serum and urine ACE2 activity was determined after 16 hours of incubation. The plates were read using a fluorescence plate reader Tecan Infinite 200 (TECAN Instruments) at an excitation wavelength of 320 nm and an emission wavelength of 400 nm. Results were expressed as RFU (Relative Fluorescent Units) per µl of sample and per hour (RFU/µl/hr).

### Kidney ACE2 Enzymatic Activity

Kidney cortex samples were homogenized in a buffer consisting of 50 mM HEPES, pH 7.4, 150 mM NaCl, 0.5% Triton X-100, 0.025 mM ZnCl_2_, and 0.1 mM Pefabloc SC Plus (Roche) and EDTA-free protease inhibitor cocktail tablet (Roche) and clarified by centrifugation at 14,000×*g* for 10 min at 4°C. After measuring protein concentration by Micro BCA assay kit (Thermo Scientific), tissue samples were diluted in a buffer containing 100 mM Tris-HCl, 600 mM NaCl, 10 µM ZnCl2, pH 7.5 and 100 µM captopril, 5 µM amastatin, 5 µM bestatin (all from Sigma-Aldrich), and 10 µM Z-Pro-prolinal (Enzo Life Sciences). To each well, 40 µl of a diluted tissue sample (0.5 µg of total renal protein) was added, along with 10 µl of buffer (with or without an specific ACE2 inhibitor, MLN-4760), and the reaction was initiated by the addition of 50 µl of the substrate (5 µmol/l, final concentration). Kidney cortex ACE2 activity was determined after 4-hour incubation at 37°C. The plates were read as described above. Experiments were carried out in duplicate for each data point. Results after subtraction of the inhibition value were expressed as RFU (Relative Fluorescent Units) per µg of protein and per hour (RFU/µg/hr).

### ACE2 Protein Expression

The tissues were snap frozen in liquid nitrogen and stored at −80°C until use. Frozen samples from kidney cortex tissue were homogenized in lysis buffer, containing 50 mM Tris-HCl pH 6.8, 100 mM NaCl, 1% Triton X-100, 20 mM EDTA, 1 mM DTT and 0.1 mM Pefabloc SC Plus (Roche) and EDTA-free protease inhibitor cocktail tablet (Roche). Homogenates were clarified by 14,000×*g* for 10 min at 4°C. Protein concentrations in the supernatant were determined by the Micro BCA assay kit (Thermo Scientific) by using BSA as standard.

Western blot analysis was performed using 15 µg of protein for each sample in a 7.5% SDS-polyacrylamide gels and transferred onto PVDF membranes (GE Healtcare Life Science). The membranes were blocked with 5% skimmed milk in TBS containing 0.2% Tween-20 for 1 h at room temperature. The membranes were then incubated for 18 h at 4°C with 1∶1000 dilution of antibody against human ACE2 (rabbit polyclonal IgG, Abcam), followed by incubation with 1∶3000 dilution of anti-rabbit secondary antibody conjugated to horseradish peroxidase (Sigma). Proteins were detected by enhanced chemiluminescence (ECL Plus, GE Healthcare Life Science) on Hyperfilm ECL (GE Healthcare Life Science), according to the manufacturer’s instructions. To control for protein loading, all membranes were probed with a monoclonal anti-β-actin antibody (mouse ascites fluid; Sigma). Densitometric analysis of the protein bands was performed using the Image J software and results were expressed as ACE2 to β-actin ratio.

### Statistical Analysis

Statistical analyses between groups were performed by ANOVA test (SPSS 12.0 for Windows). The Mann-Whitney nonparametric test was used for group-to-group comparison. Bivariate association between quantitative variables was assessed by the Spearman’s correlation coefficient. Statistical significance was defined when p≤0.05. Data are expressed as mean±SEM.

## Results

### General Findings

Blood glucose was increased about 5-fold in NOD diabetic mice (Diabetic) as compared to NOR control (Control) mice of similar age (Table 1). In NOD diabetic mice treated with insulin (Diabetic+INS), glucose levels were markedly decreased as compared with non-treated diabetic mice. In fact, the glucose levels achieved in Diabetic+INS mice, both at early stage and late stage, were not significantly different from those observed in non–diabetic controls.

In early stage group, a marked increase in the right kidney weight was observed in Diabetic mice as compared to Control mice. The administration of insulin to NOD diabetic mice reduced the increase in the kidney weight but not to the level of control mice. Therefore, the kidney/body weight ratio was increased in Diabetic as compared to control mice. This increase in the kidney/body weight ratio was decreased by the administration of insulin to NOD diabetic mice (Table 1).

In the late stage, the kidney/body weight ratio was also significantly increased in Diabetic mice as compared to control mice. However, there were no significant differences in the right kidney weight between diabetic mice and control NOR mice likely because in this group prolonged uncontrolled diabetes and its associated morbidity had resulted in a decrease in total body weight as well. Insulin administration to diabetic mice, increased both renal weight and the kidney/body weight ratio as compared to control NOR mice but not when compared to untreated NOD.

**Figure 1:**
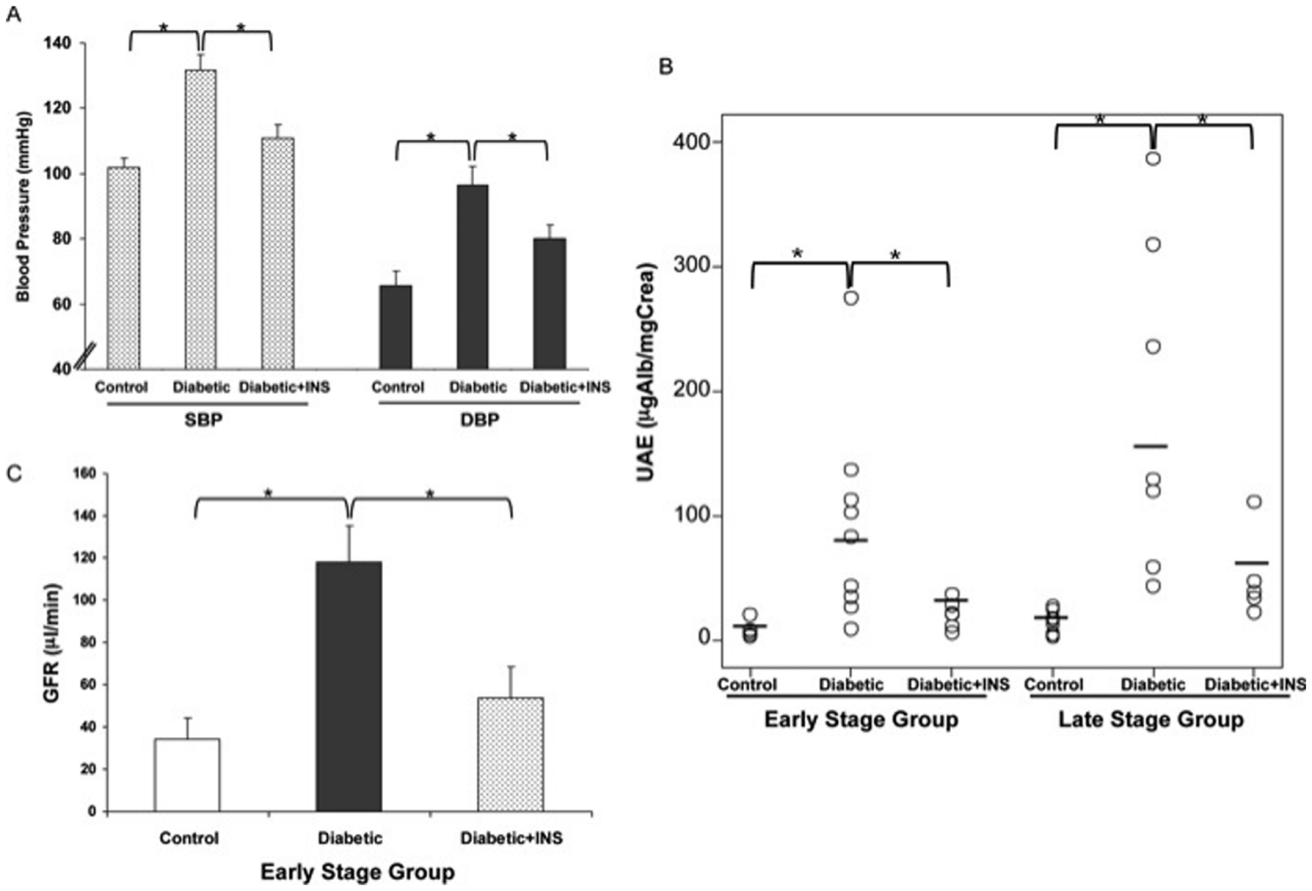
Blood pressure and kidney function parameters in non-treated non-obese diabetic mice (Diabetic), NOD-insulin treated mice (Diabetic+INS) and non-obese resistant non-diabetic mice (Control). A: At the end of the early stage study, systolic and diastolic blood pressure measured by tail-cuff method in Diabetic, Diabetic+INS and Control mice. B: Urinary albumin/creatinine ratio of Diabetic, Diabetic+INS and Control mice at both study stages. C: Glomerular filtration rate measured by inulin in Diabetic, Diabetic+INS and Control mice studied at early stage of diabetes. *P≤0.05.

At the end of the early stage follow-up, systolic blood pressure (SBP), diastolic blood pressure (DBP), and heart rate were measured in conscious animals one week before the sacrifice. SPB and DBP were significantly higher in NOD as compared to control NOR mice. The administration of insulin to Diabetic mice normalized SBP and DBP (Figure 1A). There were no differences in heart rate between groups.

### Kidney Function

There were significant differences in UAE between the three groups studied. In early stage, Diabetic mice, UAE was approximately 10-fold higher than in control mice. Insulin administration to Diabetic mice significantly decreased UAE. In late stage, Diabetic mice, UAE was approximately 21-fold higher than in controls. Insulin administration to Diabetic mice significantly decreased UAE (Figure 1B).

GFR was significantly increased in Diabetic mice as compared with control NOR mice (118±17.16 µL/min vs. 34.1±10.03, p<0.05), and the administration of insulin to Diabetic mice significantly decreased GFR (53.9±14.72, p<0.05) (Figure 1C).

### Renal Histological Findings

**Figure 2:**
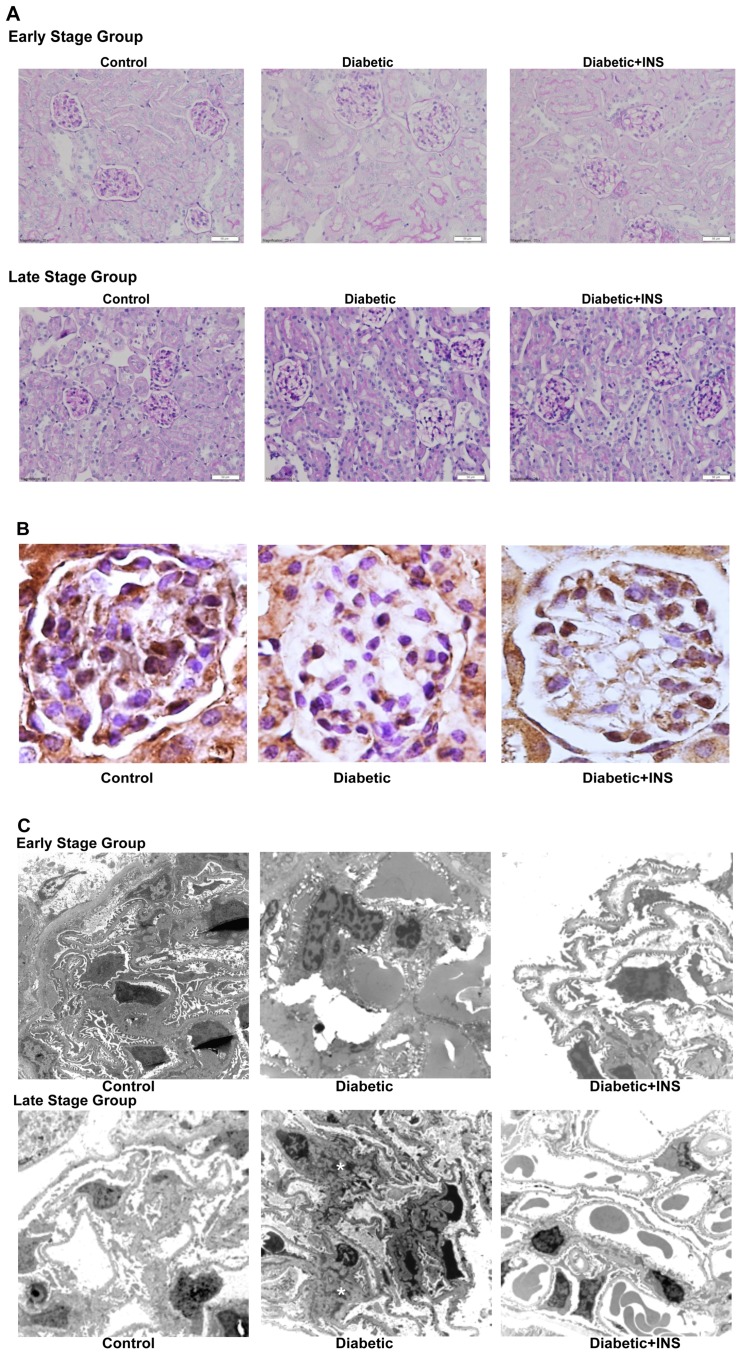
**A. Representative micrographs of periodic acid–Schiff (PAS)-stained kidney sections.** Upper panel: Diabetic, Diabetic-INS early stage of diabetes and Control mice. B: Lower panel: Diabetic, Diabetic-INS late stage of diabetes and Control mice. **B. Podocyte number in Diabetic, Diabetic-INS, and Control mice.** Representative photomicrograph depicting glomerular WT-1 staining in glomeruli from Diabetic, Diabetic-INS mice at late stage of diabetes and Control mice. Original magnification X400. **C. Ultrastructural studies in non-treated non-obese diabetic mice, Diabetic-insulin treated mice and Controls.** Upper panel: Diabetic, Diabetic-INS early stage of diabetes and Control mice. Lower panel: Diabetic, Diabetic-INS late stage of diabetes and Control mice. Glomeruli from late stage NOD Diabetic mice (lower middle panel) showed increased mesangial expansion due to increased mesangial matrix increase (see asterisks) as compared with Control mice. Insulin administration prevented mesangial expansion in NOD Diabetic mice. Original magnification, X4600.

Kidneys of early and late stage Diabetic mice showed no gross abnormalities and normal architecture of the cortex and medulla, comparable to controls by conventional light microscope. Glomeruli appeared normal and showed no sclerosis. The administration of insulin for 21 or 40 days did not alter the kidney light microscope architecture in Diabetic mice (Figure 2A).

The morphometric findings in kidneys from control, early stage Diabetic mice, and late stage Diabetic mice treated and untreated with insulin are summarized in Table 2. Using computer-linked image analysis, the glomerular tuft area was significantly increased in early stage Diabetic mice as compared to the Control group. The administration of insulin prevented the increase of the glomerular tuft area (Table 2). In the late stage, glomerular tuft area was not significantly different in control, Diabetic and Diabetic+INS mice. This is consistent with the lack in increase in kidney weight in the Diabetic mice of this age group where total body weight was reduced probably as a result of prolonged uncontrolled diabetes (Table 1).

**Table 2:** Morphometric analysis for NOD diabetic mice (Diabetic), NOD diabetic mice treated with insulin (NOD+INSULIN) and NOR control mice (Control).

	Early stage of diabetes			Late stage of diabetes		
	Control	Diabetic	Diabetic+INS	Control	Diabetic	Diabetic+INS
*N*	8	9	7	8	8	6
Glomerular tuft area (µm^2^)	6237.60±226.2	8323.79±270.98*^‡^	6539.19±196.44	6896.86±346.69	7086.47±201.51	8256.17±542.04
Glomerular cellularity (cells/glom)	27.57±0.91	31.06±1.31	30.14±1.05	33.09±1.60	34.38±2.06	29.29±1.98
Podocyte number (%pod/glom)	46.79±1.94	39.41±2.84	42.10±2.50	36.31±2.43	26.66±1.56*^‡^	33.50±2.38

Data are expressed as means± s.e.

p≤0.05*Diabetic vs Control.

p≤0.05^‡^Diabetic vs. Diabetic+INS.

The mesangial matrix index derived from light mycroscopy was not different between Diabetic mice as compared to control NOR mice (early: 44.76%±2.54 vs 41.84±1.57, p = 0.35//late: 40.17±1.57 vs 41.84±1.57, p = 0.55). The administration of insulin for 21 or 40 days did not alter mesangial index (early: 43.79±1.63//late: 38±1.13).

Diabetic mice at early and late stage diabetes showed higher glomerular cellularity as compared to NOR controls but this did not reach statistical significance (Table 2). In Diabetic mice insulin treatment slightly reduced the total cell number per glomerulus.

The proportion of glomerular cells that were identified as podocytes was non-significantly decreased in early stage Diabetic mice as compared to control NOR animals (Table 2). Only in late stage Diabetic mice, the decrease in podocyte number was markedly reduced as compared to control NOR mice (26.66%±1.56 vs. 36.31±2.43, p<0.05). The administration of insulin for 40 days almost completely prevented the decrease of podocyte number as compared with non-treated Diabetic mice (33.5%±2.38, p<0.05) (Figure 2B).

### Electron Microscopy

Ultrastructural analysis of glomeruli from late stage NOD diabetic mice showed increased mesangial volume as compared to NOR control mice (0.290±0.023 vs. 0.134±0.021, p<0.05). Insulin administration prevented this mesangial expansion in NOD diabetic mice (0.127±0.025, p<0.05). We did not observe ultrastructural changes in early stage NOD diabetic mice as compared to control NOR mice (Figure 2C).

Glomerular basement membrane (GBM) thickness was increased in glomeruli from late stage NOD diabetic mice as compared to NOR controls (191.22±3.05 nm vs. 162.95±2.35). Insulin administration prevented GBM increase in NOD diabetic mice (167.86±4.67). We did not observe any increase in GBM in early stage NOD diabetic mice as compared to NOR controls.

### ACE2 Enzyme Activity in Serum and Urine

**Figure 3:**
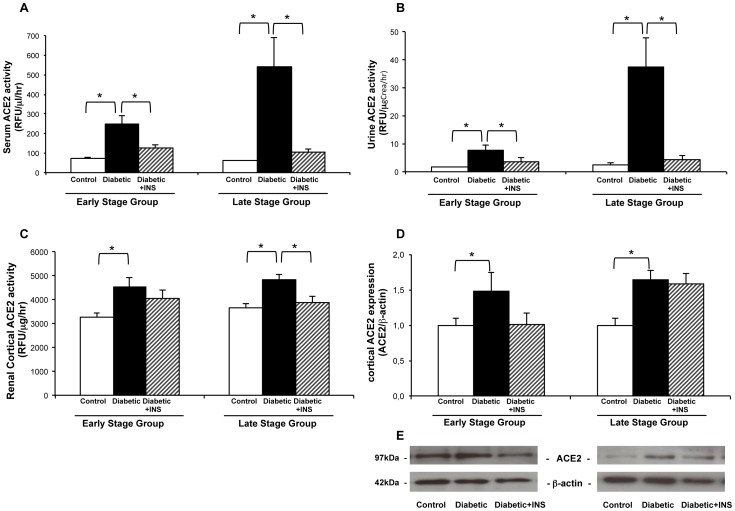
ACE2 activity in serum (A), urine (B) and kidney cortex (C) from non-treated non-obese diabetic mice (Diabetic), NOD-insulin treated mice (Diabetic+INS) and non-obese resistant non-diabetic mice (Control) (A, B, C). ACE2 protein expression measured by western blot of protein preparations from kidney cortex lysates in Diabetic, Diabetic+INSand Control mice (D). Immunoblot analysis of ACE2 in kidney cortex tissue (E). Shown is representative immunoblot of ACE2 protein in kidney cortex from Control, Diabetic, and Diabetic+INS mice, at early and late stage of diabetes. *P≤0.05.

The enzymatic activity of ACE2 was measured in serum and urine in early and late stage Diabetic mice (Figure 3A). Serum from Diabetic mice showed increased ACE2 enzyme activity as compared to controls. In both, early and late stage of diabetes, enzyme activities in serum were significantly increased (*early*:249.8±39 vs. 71.54±4.26 RFU/µL/h; *late*: 543.53±145.9 vs. 60.91±2.46, p<0.05). After insulin treatment of NOD diabetic mice ACE2 enzyme activity dramatically decreased near to the control levels, at both study points (*early*:126.7±14.8; *late*:106.6±15.62, p<0.05).

Spot urine samples obtained from early and late stage NOD mice were used to determine ACE2 enzymatic activity (Figure 3B). As described for serum samples, we found increased ACE2 activity in Diabetic mice as compared to controls, at early stage (7.82±1.63 vs. 1.63±0.07 RFU/µgCrea/hr, p<0.05) and also at late stage (37.49±10.27 vs. 2.39±0.71, p<0.05). As shown for serum samples, insulin treatment markedly decreased ACE2 enzyme activity near to the levels of control NOR in both early and late stage (*early*:3.74±1.31, p<0.05; *late*:4.2±1.69, p<0.05).

### ACE2 Enzyme Activity in Kidney Cortex

Kidney cortical ACE2 enzymatic activity was quantified in both early and late stage groups (Figure 3C). ACE2 enzyme activity was significantly increased in renal cortex from both early and late stage Diabetic mice as compared to non-diabetic controls (*early*:4512±426 vs. 3285±167, RFU/µg protein/hr; *late*:4852±219 vs. 3637±206, p<0.05). Insulin treatment significantly decreased ACE2 enzymatic activity in late stage Diabetic mice (3859±268, p<0.05). However, no significant changes were observed in early stage NOD mice treated with insulin.

### ACE2 Protein Expression in Renal Cortex

ACE2 protein expression was increased in renal cortex from both early and late stage Diabetic mice as compared to non-diabetic controls (*early*: 1.48±0.26 vs. 1±0.10, ACE2/β-actin; *late*:1.64±0.14 vs. 1±0.09, p≤0.05) (Figure 3D). Insulin pellets slightly decreased ACE2 protein expression in early stage and late stage NOD-treated mice, as compared to NOD non-treated mice without statistically significance (*early*: 1.01±0.16; *late*: 1.59±0.15, p = NS).

### Correlation between Serum and Urine ACE2 and Kidney Function Parameters

**Figure 4:**
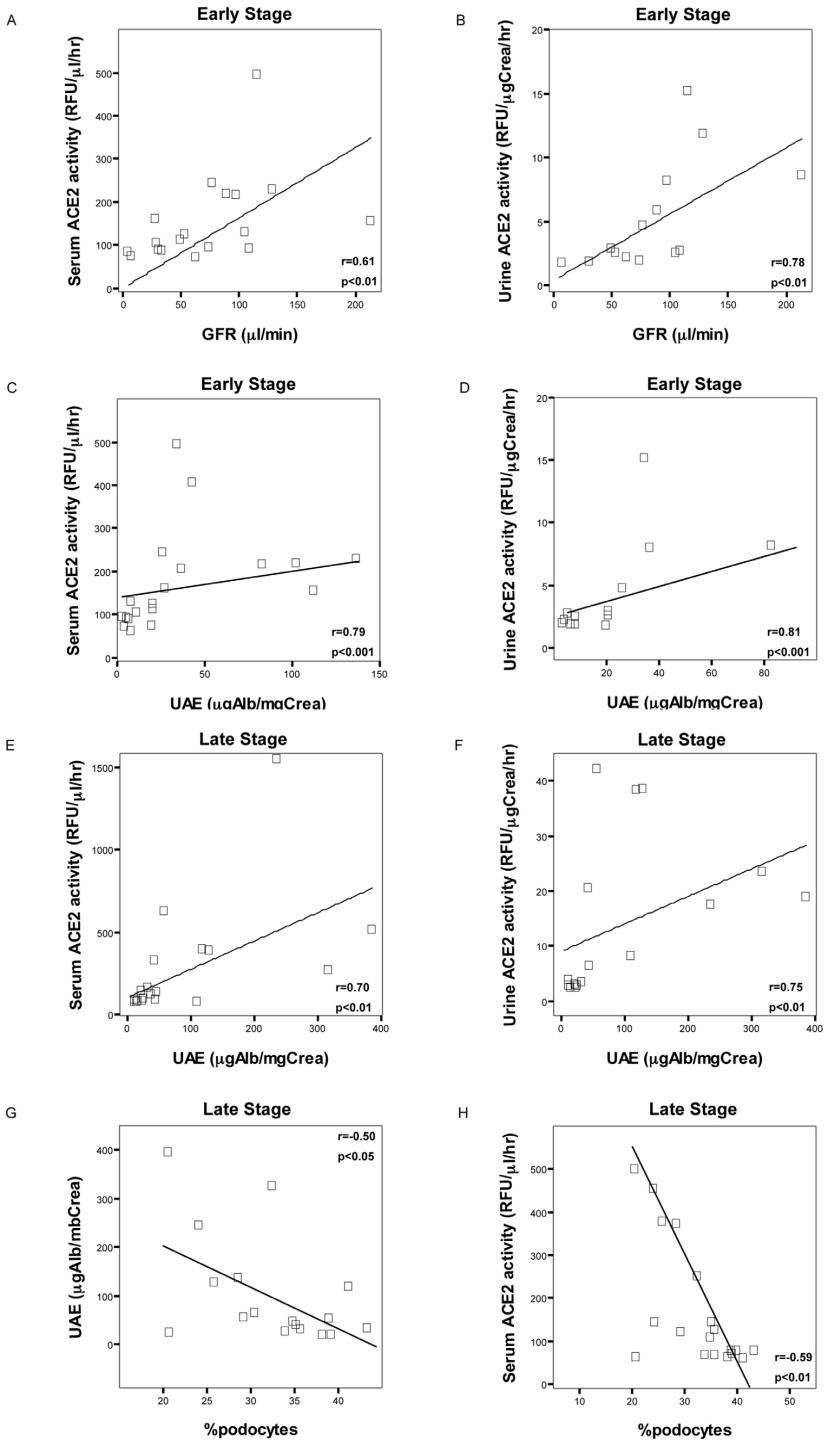
Correlation between serum and urine ACE2 and kidney function parameters. ACE2 activity in serum (A) and urine (B) directly correlated with glomerular filtration rate in early stage of non-treated non-obese diabetic mice (Diabetic), NOD-insulin treated mice (Diabetic+INS) and non-obese resistant non-diabetic mice (Control). In addition, ACE2 activity in serum (C, E) and urine (D, F) also directly correlated with urinary albumin excretion at early (C, D) and late (E, F) stage of diabetes in NOD mice, Diabetic+INS and Control mice. Furthermore, podocyte number indirectly correlated with urinary albumin excretion and serum ACE2 activity in late stage of Diabetic, Diabetic+INS and Control mice (G, H).

Serum ACE2 activity from early stage Diabetic mice, Diabetic-INS treated mice and NOR controls, had a significant positive correlation with GFR (r = 0.84, p<0.001). In concordance with this finding, urine ACE2 activity also showed a positive correlation with GFR (r = 0.84, p<0.001). Furthermore, in early stage Diabetic mice, there were a direct correlation between serum and urine ACE2 activity and UAE (r = 0.79/r = 0.81, p<0.001) (Figure 4A–D). In addition, in late stage group, there were a direct correlation between serum and urine ACE2 activity and UAE (r = 0.72/r = 0.79, p<0.001) (Figure 4). Furthermore, in the late stage group, there was an indirect correlation between serum ACE2 activity and the percentage of podocytes per glomerular tuft cross-sections (r = −0.59, p<0.05). In addition, in the late stage group, UAE showed a negative correlation with the percentage of podocytes (r = −0.50, p<0.05) (Figure 4E–H).

## Discussion

This study shows that in the NOD model of type 1 diabetes, there is an increase in GFR and glomerular size early in the course of the disease that mimics the renal hypertrophy and hyperfiltration characteristic of early nephropathy in human type 1 diabetes. Insulin prevented the development of hyperfiltration as shown by normalization of GFR, and a decrease in kidney weight and glomerular size in the early stage disease group. Of interest also is the finding that blood pressure was increased in the early stage disease in the Diabetic group and after insulin it was markedly reduced. Overt hypertension is not an early finding in patients with type 1 diabetes but in those destined to develop microalbuminuria, hypertension can manifest as nocturnal hypertension while daytime blood pressure is still normal [28]. Since most type 1 diabetic patients are treated with insulin, it is reasonable to speculate, extrapolating from our findings that insulin lowers blood pressure in the NOD mice, that hypertension in such patients could be masked by the treatment with insulin to control diabetes. The vasodilatory action of insulin is well known [29] but its potential hypotensive effect has been the subject of debate and controversy over the years [29], [30], [31]. We surmise, based on our experimental results, that insulin directly may exert an important hypotensive action when given to type 1 diabetes patients. The universal use of insulin could be a reason why overt hypertension is not commonly manifested early on in patients with type 1 diabetes.

Our study also shows that serum and urine ACE2 activity is increased in the NOD mouse model of diabetes at early and late stages of the disease. Interestingly, glycemic control by insulin administration prevented the diabetes-induced increase in the serum and urine ACE2 activity in the Diabetic mice. ACE2 activity and expression was also increased in kidney cortex in both early stage and late stage NOD Diabetic mice. Colucci et al. found decreased ACE2 protein expresion in kidney cortex from Diabetic mice, however, these authors did not measure ACE2 enzymatic activity. Also in Colucci study animal age and diabetes duration is not stated, whereas in our study animals were followed for 21 and 40 days after the diagnosis of diabetes [32]. Thus, it is possible that differences in ACE2 protein content between both studies reflect differences in age and disease evolution over time.

Previous studies have shown that ACE2 activity in kidney cortex was increased in two different rodent models of diabetes, the STZ and the db/db diabetic mice [16], [33], but serum and urine ACE2 activity was not reported in these previous studies. ACE2 cleaves AngI to form Ang-(1–9) and AngII to form Ang-(1–7) [13], [14]. ACE2 thus prevents AngII accumulation while favoring Ang-(1–7) formation. Ang-(1–7) has vasodilatory, natriuretic, and antiproliferative actions [34]. Its enhanced formation may have a beneficial effect and counterbalance the deleterious actions of AngII in terms of kidney damage. Thus, increased ACE2 activity in serum, urine, and renal cortex might suggest a compensatory mechanism to downregulate the circulating and renal AngII accumulation in the diabetic kidney, suggesting a potential mechanism to adapt to diabetes associated AngII overactivity.

Insulin administration prevented albuminuria, glomerular enlargement and alterations in GFR as early kidney alterations in the NOD Diabetic mice. Our findings are consistent with the notion that both, abnormal renal function and renal enlargement can regress after a period of strict glycemic control in patients with type 1 diabetes [5]. Our study also showed that good glycemic control avoids renal lesions in late stage of diabetes in NOD mice, when ultrastructural alterations are already observed. However, we did not evaluate the regression of renal pathology. Previous studies performed in STZ-induced diabetic rats showed that normalization of blood glucose by insulin reversed established glomerular hyperfiltration, renal hypertrophy in hyperglycemic diabetic rats [35]. Furthermore, continuous subcutaneous insulin therapy instituted after the development of early glomerular pathology was effective in arresting the disease process [36]. At cellular level, podocytes need to be insulin sensitive to maintain cellular integrity as recently published. Furthermore, these actions have been associated with the modulation of VEGF production through the insulin signal [37].

Regarding human studies, several publications have showed that early intensive diabetes therapy in the course of type 1 diabetes prevents renal damage progression as compared to patients treated with conventional diabetic therapy [38], reinforcing the importance of early and intensive diabetic control among patients with type 1 diabetes.

Several studies have been focused in the study of ACE2 alterations within the diabetic kidney [15], [16], [33], [39], [40], [41], [42], [43], [44], and these have found increased ACE2 in kidney cortex from the db/db, STZ and Akita mouse models of diabetes [16], [33], [45]. These results are consistent with our findings in NOD diabetic mice. However, urine and serum ACE2 activity and its correlation with renal function in diabetic mice have not been previously studied. Interestingly, our study demonstrates that serum and urine ACE2 activity is increased in NOD diabetic mice, and correlates with increased UAE and increased GFR, as early markers of kidney involvement in diabetes. Our findings suggest that within the RAS system, ACE2 activity is early altered in the natural evolution of the diabetic kidney. Normalization of glycemia prevents renal alterations such as glomerular enlargement and hyperfiltration, and these effects are associated with prevention of ACE2 elevation. Insulin administration decreases ACE2 activity in kidney cortex from late stage Diabetic mice, but does not reduce ACE2 protein abundance. We cannot fully explain this discrepancy, but these results might suggest that ACE2 activity should be measured when studied this enzyme. Soro-Paavonen et al. recently reported on the levels of ACE2 activity in patients with type 1 diabetes. They demonstrated that ACE2 activity is increased in male and female with type 1 diabetes and altered kidney disease or other cardiovascular complications such as coronary heart disease [17]. Serum ACE2 activity positively correlated with systolic blood pressure and diabetes duration among male type 1 diabetic patients. Furthermore, ACE2 activity was also increased in men with type 1 diabetes and microalbuminuria. Consistent with these findings, serum and urine ACE2 activity directly correlated with UAE in the NOD diabetic mice (Figure 4). These results suggest that serum and urine ACE2 activity may be increased as a renoprotective mechanism and that this enzyme is already activated in an early stage of diabetic kidney disease, when pathological kidney lesions are not observed yet.

Circulating ACE2 activity has been previously studied in STZ-induced diabetic mice and rats [46], [47]. Tikellis and colleagues reported a 2-fold increase in circulating ACE2 in STZ-induced diabetic male mice [46]. In concordance, a recent study found that early onset diabetes increased ACE2 activity 9-fold in female and 3-fold in male mRen2.Lewis; the greater magnitude change in ACE2 likely due to the lower circulating activity in control females [47]. Our ACE2 activity studies also reveal a 10-fold higher circulating ACE2 in female NOD diabetic mice as compared to control females. Interestingly, as kidney disease progresses, circulating ACE2 activity in diabetic mice was further increased. The increase in circulating ACE2 may attenuate a greater increase in AngII in diabetes mellitus [48]. Indeed, the exacerbation of diabetic injury with an ACE2 inhibitor or in ACE2 knockout mice may reflect, in part, the loss of the circulating enzyme and the ability to effectively buffer circulating AngII [15], [45].

Early loss of podocytes in the diabetic rat and mouse models implicates podocyte damage in the pathogenesis of diabetic nephropathy. It may be that a certain threshold of podocyte damage must be achieved before sclerosis occurs [49], [50]. Siu et al. found that podocyte density and apparent glomerular podocyte number are substantially reduced in rats and mice injected with STZ, a model of Type 1 diabetes mellitus, quite early after initiation of diabetes. Reduction in apparent podocyte number and density occurred as early as 2 weeks after STZ injection, and appeared to worsen by 6 and even further by 8 weeks after STZ administration [50]. Interestingly, insulin treatment had only a modest effect in maintaining podocyte number, despite inducing a marked improvement in fasting blood glucose. Our study is the first to show decreased podocyte number in NOD Diabetic mice, and moreover, we found that insulin administration prevented the podocyte decrease in NOD diabetic mice. This suggests that tight hyperglycemic control, in a model of autoimmune diabetes that mimics human type 1 diabetes, contributes in part to the renoprotective actions of the insulin by preventing the deleterious effect of high glycemic load. Also, some other actions unrelated to the tight glycemic control may contribute to the prevention of podocyte loss in insulin-treated diabetic animals. Recent evidence suggests that insulin has multiple cellular functions on podocytes. Drapeau et al. demonstrated that SHP-1 binds with IR-β, preventing the insulin signaling pathway on Akt and ERK phosphorylation. Inhibition of SHP-1 restored insulin action and prevented podocyte apoptosis induced by HG levels [51]. These results suggest that several mechanism might be involved in the antiapoptotic effect of insulin within the kidney.

In summary, NOD diabetic mice develop features of early kidney disease including a marked increase in GFR that resemble those seen early in type 1 diabetes. Moreover, the NOD Diabetic mice develops hypertension early on and this alteration, like the renal hyperfiltration, can be prevented by insulin administration. This study also shows that serum and urine ACE2 activity is increased in the NOD mouse model of diabetes starting at an early stage of the disease. Insulin administration prevented the diabetes-induced increase in the serum and urine ACE2 activity in the NOD mice.
