# Correction: Effect of Insulin on ACE2 Activity and Kidney Function in the Non-Obese Diabetic Mouse

**DOI:** 10.1371/annotation/2a7b69d2-a049-448e-b584-51e4c1e8a50d

**Published:** 2014-01-28

**Authors:** Marta Riera, Eva Márquez, Sergi Clotet, Javier Gimeno, Heleia Roca-Ho, Josep Lloreta, Nuria Juanpere, Daniel Batlle, Julio Pascual, María José Soler

The affiliation of the fifth author is incorrect. Heleia Roca-Ho is not affiliated with institution number 2, but rather with institution number 1, Department of Nephrology, Hospital del Mar-IMIM, Barcelona, Spain.

